# Impact of early personal‐history characteristics on the Pace of Aging: implications for clinical trials of therapies to slow aging and extend healthspan

**DOI:** 10.1111/acel.12591

**Published:** 2017-04-12

**Authors:** Daniel W. Belsky, Avshalom Caspi, Harvey J. Cohen, William E. Kraus, Sandhya Ramrakha, Richie Poulton, Terrie E. Moffitt

**Affiliations:** ^1^ Department of Medicine Duke University School of Medicine Durham NC USA; ^2^ Social Science Research Institute Duke University Durham NC USA; ^3^ Center for the Study of Aging and Human Development Duke University Durham NC USA; ^4^ Department of Psychology and Neuroscience Duke University Durham NC USA; ^5^ Department of Psychiatry and Behavioral Sciences Duke University School of Medicine Durham NC USA; ^6^ Center for Genomic and Computational Biology Duke University Durham NC USA; ^7^ MRC Social, Genetic, and Developmental Psychiatry Centre Institute of Psychiatry, Psychology, and Neuroscience King's College London London UK; ^8^ Dunedin Multidisciplinary Health and Development Research Unit Department of Psychology University of Otago Dunedin New Zealand

**Keywords:** biological aging, geroscience, geroprotector, healthspan, early‐life, personal history characteristics

## Abstract

Therapies to extend healthspan are poised to move from laboratory animal models to human clinical trials. Translation from mouse to human will entail challenges, among them the multifactorial heterogeneity of human aging. To inform clinical trials about this heterogeneity, we report how humans’ pace of biological aging relates to personal‐history characteristics. Because geroprotective therapies must be delivered by midlife to prevent age‐related disease onset, we studied young‐adult members of the Dunedin Study 1972–73 birth cohort (*n* = 954). Cohort members’ Pace of Aging was measured as coordinated decline in the integrity of multiple organ systems, by quantifying rate of decline across repeated measurements of 18 biomarkers assayed when cohort members were ages 26, 32, and 38 years. The childhood personal‐history characteristics studied were known predictors of age‐related disease and mortality, and were measured prospectively during childhood. Personal‐history characteristics of familial longevity, childhood social class, adverse childhood experiences, and childhood health, intelligence, and self‐control all predicted differences in cohort members’ adulthood Pace of Aging. Accumulation of more personal‐history risks predicted faster Pace of Aging. Because trials of anti‐aging therapies will need to ascertain personal histories retrospectively, we replicated results using cohort members’ retrospective personal‐history reports made in adulthood. Because many trials recruit participants from clinical settings, we replicated results in the cohort subset who had recent health system contact according to electronic medical records. Quick, inexpensive measures of trial participants’ early personal histories can enable clinical trials to study who volunteers for trials, who adheres to treatment, and who responds to anti‐aging therapies.

## Introduction

The prevalence of many chronic diseases increases steeply with advancing chronological age (Belsky *et al*., 2015a). Thus, aging itself can be considered a leading disease risk factor (Kaeberlein, 2013; López‐Otín *et al*., 2013). This observation implies that interventions to slow biological aging could delay all age‐related diseases simultaneously (Kirkland, 2013), reducing late‐life multimorbidity (Barnett *et al*., 2012) and extending years lived free of disease and disability, called ‘healthspan’ (Burch *et al*., 2014). The aging global population makes development of healthspan‐extending interventions a public health priority (Harper, 2014). Researchers pioneering geroprotective therapies in animals appear poised to deliver these therapies to human trials (de Cabo *et al*., 2014; Longo *et al*., 2015). But human translation of therapies to slow the biological process of aging will face challenges (Moffitt *et al*., 2016; Moskalev *et al*., 2016).

One likely challenge to translation from mouse to man is that free‐living humans are heterogeneous as compared to laboratory‐based model organisms. In contrast to genetically identical animals living under uniform laboratory conditions, humans’ pace of biological aging may be sped or slowed by personal‐history characteristics that accumulate from early life (Kirkwood & Austad, 2000; Gavrilov & Gavrilova, 2004; Gladyshev, 2016). As examples, familial longevity (Perls & Terry, 2003; Atzmon *et al*., 2004), childhood social disadvantage and adverse experiences (Felitti *et al*., 1998; Hayward & Gorman, 2004), and childhood traits including poor health and low intelligence (Case *et al*., 2005; Calvin *et al*., 2011) are able to forecast later‐life disease and mortality.

**Figure 1:**
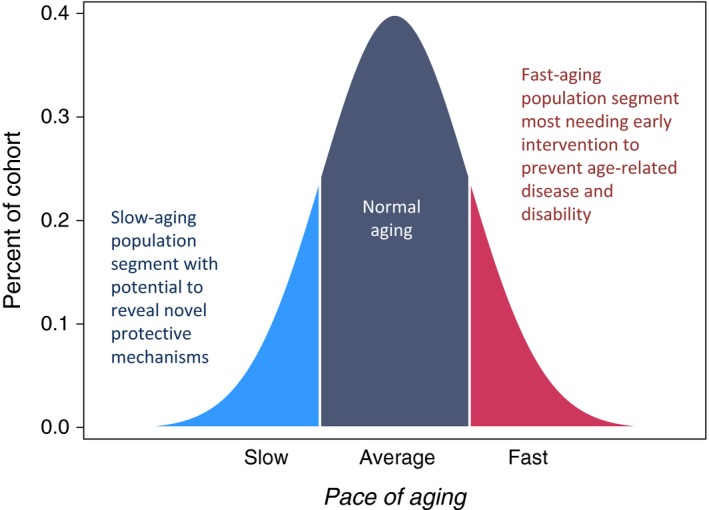
Subgroups of normally distributed human pace of aging relevant to design of trials for healthspan‐extension therapies. Pace of aging is the rate of coordinated decline in the integrity of bodily systems occurring with advancing chronological age. We showed that variation in the pace of aging could be quantified already in individuals still too young to have age‐related disease by tracking changes in biomarkers of organ system functioning over time (Belsky *et al*., 2015a). We earlier reported that individuals whose physiology changed more slowly with the passage of chronological time (light blue segment of distribution) experienced better physical and cognitive functional outcomes in aging, and also showed fewer subjective signs of aging. The opposite was true of those whose physiology changed more rapidly (red segment of distribution). Both slower‐aging and faster‐aging population segments are needed in research to develop healthspan‐extension therapies. Slower‐aging populations may provide clues to novel therapeutic targets. Faster‐aging populations are those who therapies must benefit.

Studying personal‐history characteristics that may influence the pace of biological aging is important, at least in part, because personal characteristics have the capacity to impact translation from preclinical healthspan models to humans (Guarente, 2014; Pitt & Kaeberlein, 2015). First, personal‐history characteristics related to the pace of aging are known to influence the propensity to volunteer for trials, as well as the likelihood of completing protocols and adhering to treatment regimens. Second, personal histories may influence response to treatment. To demonstrate the benefit of potential geroprotective treatments for improving public health, at least some trials will need to recruit individuals with personal histories of adverse exposure to effectively represent populations most needing healthspan‐extending therapies (Fig. 1). Likewise, advantaged personal histories could identify individuals who are already aging slowly, and are unlikely to benefit from treatment. Third, randomized clinical trials are obliged to register in advance participant characteristics to be analyzed as potential moderators of treatment outcome. Trials will need to collect data on such characteristics. Information about personal‐history characteristics that influence the pace of human aging could thus improve trial design and registration.

We tested the hypothesis that worse scores on personal‐history characteristics would predict a faster pace of biological aging among 954 members of the 1972–73 Dunedin (New Zealand) birth cohort, who have now reached approximately the midpoint of the contemporary human lifespan. We studied a midlife cohort because this is the age group for whom age‐delaying therapies can still delay the onset of chronic disease. For example, this midlife strategy was adopted by the first human trial of caloric restriction as an intervention designed to extend healthspan (Ravussin *et al*., 2015).

We measured the pace of biological aging from changes in 18 biomarkers of cohort members’ cardiovascular, metabolic, endocrine, pulmonary, hepatic, renal, immune, and periodontal systems. These 18 biomarkers were measured repeatedly across 12 years, at three time points when the cohort members were aged 26, 32, and 38 years. We use these data to measure decline that occurs simultaneously across multiple biomarkers. By studying repeated measures of biomarkers over time, we can distinguish age‐related changes from baseline differences that reflect prior health status. By studying changes in multiple biomarkers together, we can distinguish age‐related loss of system integrity from spikes in particular biomarkers caused by acute illness. Our measure, called the ‘Pace of Aging’, operationalizes the coordinated progressive loss of integrity across bodily systems that geroscience theory specifies as the aging process (Belsky *et al*., 2015a). Measured Pace of Aging is not a cause of biological aging; it is a measurement of differences among individuals in their rate of biological aging during our 12‐year observation window. The Pace of Aging quantifies Study members’ rate of biological aging in year‐equivalent units of physiological decline occurring per chronological year. The average Study member experienced 1 year of physiological decline per each chronological year, a Pace of Aging of 1. The fastest‐aging Study members experienced more than twice this rate of change, while the slowest‐aging Study members experienced almost no change at all.

Here we report relationships of personal‐history characteristics with Pace of Aging. The six characteristics were familial longevity, childhood social class, adverse childhood experiences, and childhood health, intelligence, and self‐control. We selected these characteristics because each has an established link to morbidity and mortality (Friedman *et al*., 1993; Felitti *et al*., 1998; Atzmon *et al*., 2004; Hayward & Gorman, 2004; Case *et al*., 2005; Calvin *et al*., 2011). We focused on familial and childhood measures because of evidence that early‐life exposures increase risk for diseases that reduce healthspan (Power *et al*., 2013). Early‐life measures also have the advantage of being temporally antecedent to the biomarkers that we used to quantify Study members’ pace of biological aging; this eliminated the possibility of reverse causation. Finally, we selected early‐life characteristics that clinical trials are able to ascertain from trial participants; for example, we did not test low birthweight because it is difficult to ascertain by retrospective report. We initially measured the six personal‐history characteristics using prospective data collected during childhood waves of the study. However, knowing that few trials will have access to prospective childhood measurements, we also conducted a parallel analysis using measurements taken retrospectively, when Study members were adults.

## Results

### Personal histories predict adults’ Pace of Aging

To test whether Study members’ personal‐history characteristics were related to their Pace of Aging, we examined six characteristics. Grandparental longevity was measured as the oldest age to which any biological grandparent had survived. Childhood social class was defined from the occupations of Study members’ parents when Study members were children. Adverse childhood experiences (ACEs) were defined according to the US Centers for Disease Control and Prevention criteria (Felitti *et al*., 1998). Childhood health was measured as a combination of examinations, nurse ratings, and clinical interviews with parents (Belsky *et al*., 2015b). Childhood IQ was measured using the Wechsler Intelligence Scales for Children (Wechsler, 2003). Childhood self‐control was measured as a combination of staff observations, and parent, teacher, and children's own reports (Moffitt *et al*., 2011).

**Figure 2:**
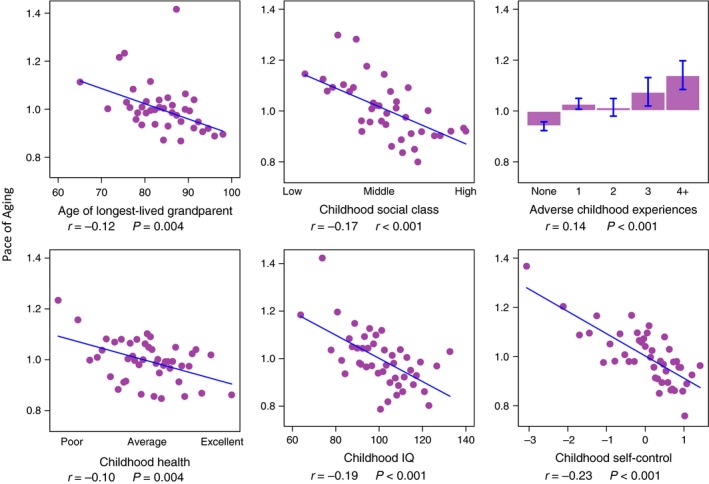
Family and childhood characteristics are associated with Study members’ Pace of Aging from age 26 to 38 years. Figure cells graph associations between six family and childhood characteristics (*x*‐axis variables) and Study members’ Pace of Aging measured from changes in 18 biomarkers measured across ages 26, 32, and 38 years (*y*‐axis). Age of longest‐lived grandparent was measured from reports by Study members’ parents. Childhood social class, exposure to adverse childhood experiences, childhood health, childhood IQ, and childhood self‐control were assessed using previously established methodology applied to archival Dunedin Study records including examinations and testing, reports by parents and teachers, clinician ratings, and direct observations. Figures show ‘binned’ scatterplots in which each plotted point reflects average x‐ and y‐coordinates for “bins” of approximately 20 Study members. Regression lines and effect size estimates were estimated from the original, unbinned data.

Study members with shorter‐lived grandparents, who grew up in lower social class homes, who experienced more ACEs, who had poorer childhood health, who scored lower on IQ tests, and who had poorer self‐control all showed evidence of accelerated biological aging during their 20s and 30s (Pearson's r range *r* = 0.10–0.23, Fig. 2).

**Figure 3:**
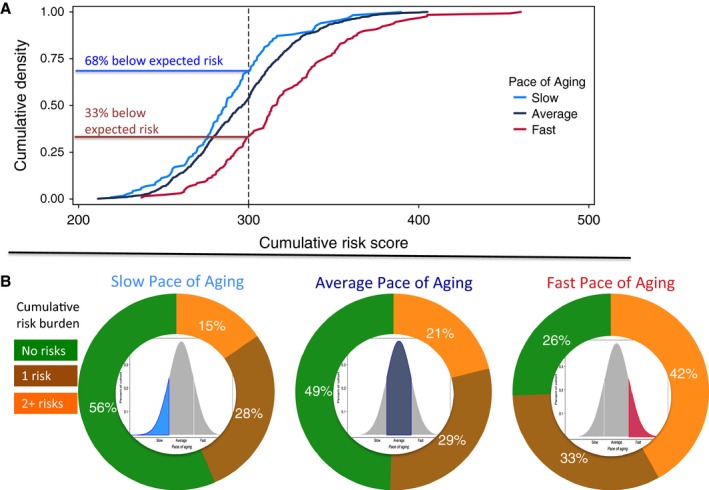
Cumulative prospectively assessed personal‐history risks in Study members with slow, average, and fast Pace of Aging. Panel A graphs density plots of cumulative risk scores for Study members with slow, average, and fast Pace of Aging. The cumulative risk score reflects total burden of risk across six personal‐history characteristics (grandparent longevity, family social class during childhood, adverse childhood experiences, childhood IQ score, childhood self‐control, and childhood health). For each characteristic, values were standardized to a T‐distribution (M = 50, SD = 10) with high scores reflecting increased risk (e.g., shorter‐lived grandparents, lower childhood social class). Standardized values were summed to calculate the cumulative risk score. Thus, the expected cumulative risk level was 300. The graph shows that two‐thirds of the slow‐aging group had below this expected level of risk. In contrast, less than one‐third of the fast‐aging group did. Panel B graphs proportions of Study members with slow, average, and fast Pace of Aging (see Fig. 1) who were classified as high risk on 0, 1, or 2 or more of the six characteristics. High‐risk classifications were for having short‐lived grandparents (no grandparent survived past age 80 years), growing up in a low social class family, exposure to four or more adverse childhood experiences, childhood IQ score ≤1 SD below the population mean (a score of 85 or below), childhood self‐control score ≤1 SD below the population mean, and childhood health score ≤1 SD below the population mean. The graph shows that most slow‐aging Study members had no high‐risk classifications. In contrast, more than 40% of the fast‐aging Study members were classified as high risk on multiple family and childhood characteristics.

Childhood risk characteristics tend to cluster within individuals, and their accumulation is known to influence later outcomes (Evans *et al*., 2013). Therefore, we tested whether accumulations of personal‐history risks differentiated rapidly aging Study members from their slower‐aging peers. To calculate a cumulative risk score, personal‐history measures were standardized to a T‐distribution (mean = 50, SD = 10) and summed. (A Study member with average levels of all six risk characteristics would have a cumulative risk score of 50 × 6 = 300.) Study members with higher cumulative personal‐history risk experienced faster Pace of Aging as compared to peers with a lower cumulative risk (*r* = 0.27, *P* < 0.001). Fig. 3 shows how cumulative risk differed between slower‐ and faster‐aging Study members. For graphing purposes, in Fig. 3, Study members were divided into three groups based on Pace of Aging: a slow‐aging group with Pace of Aging ≤1 SD below the mean (less than about 0.6 years of physiological change or less per chronological year; *n* = 117); an average‐aging group with Pace of Aging within 1 SD of the mean (about 0.6–1.4 years of physiological change per chronological year; *n* = 708); and a fast‐aging group with Pace of Aging ≥1 SD above the mean (more than about 1.4 years of physiological change per chronological year; *n* = 129). Most slow‐aging Study members had no high‐risk characteristics. In contrast, more than 40% of fast‐aging Study members were classified as high risk on multiple family and childhood characteristics.

### Can personal histories be measured in adults enrolling in trials of therapies to slow aging?

Clinical trials of therapies to slow aging are likely to lack prospective measures taken when enrollees were children; for example, few adults have records of their childhood IQ. Therefore, analyses were repeated using contemporaneous assessments of personal‐history characteristics taken after Dunedin Study members became adults. We classified each Study member as high or low risk on each of the personal‐history characteristics using quick, inexpensive measurements. We used adulthood reports of whether any biological grandparent had lived past age 80 years to assess familial longevity; adulthood recollections of Study members’ parents’ occupations during their childhoods to assess childhood social class; retrospective interviews to count ACE exposures; and educational attainment as a proxy for childhood IQ. To assess the adult equivalent of self‐control, we used brief ratings of adult Study members’ conscientious personality made by nurses during physical examinations. (Memories of childhood health had not been queried from Study members as adults.) Contemporaneously assessed history of cumulative risk was related to Pace of Aging; Study members with more personal‐history risk characteristics had faster Pace of Aging (*r* = 0.21, *P* < 0.001). This association was similar to, but slightly weaker than, the association between prospectively measured risk and Pace of Aging (*r* = 0.27).

**Figure 4:**
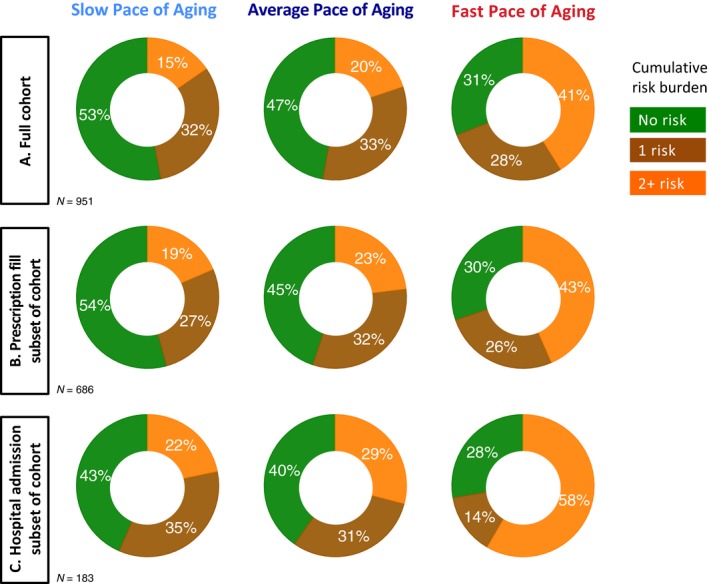
Proportions of slow, average, and fast Pace of Aging Study members classified as high risk on 0, 1, or 2 or more family and childhood characteristics based on contemporaneous assessments conducted in adulthood. Risk factors were having short‐lived grandparents (no grandparent survived past age 80 years), retrospective report by the Study member that their parents held low‐status occupations during the Study member's childhood, retrospective report of exposure to four or more adverse childhood experiences, not holding any educational credential, and being rated by an examining nurse as having low levels of the personality trait conscientiousness. Panel A graphs results for the full cohort. The pattern is the same as when risk was classified from assessments during childhood. Most slow‐aging Study members were not classified as high risk on any family or childhood characteristic. In contrast, more than 40% of the fast‐aging Study members were classified as high risk on multiple family and childhood characteristics. Panels B and C repeat the analysis for subsamples of cohort members with recent contacts with the healthcare system and who may reflect the population most accessible to recruitment into clinical trials. Panel B graphs results for Study members with a recent prescription fill. Panel C graphs results for Study members with a recent hospital admission (excluding for pregnancy‐related services).

Some clinical trials of geroprotective therapies may wish to recruit volunteers from clinical settings. To assess whether personal histories would predict pace of aging similarly in such individuals, we tested the association between personal‐history risk and Pace of Aging in Dunedin Study members who had recent contact with healthcare providers (Fig. 4). One group comprised Study members with recent prescription fills. Another group comprised Study members with recent hospital admissions. Both groups were identified via record linkage with national health system electronic medical records. Within these subgroups that may better reflect adults available for recruitment into clinical trials, more personal‐history risk characteristics continued to predict faster Pace of Aging.

Because educational attainment is only a crude proxy for childhood intelligence, and because some clinical trial samples will be relatively homogeneous in their educational attainment, we repeated our analysis substituting, in place of low education, a 2‐min, nonverbal paper‐and‐pencil test of processing speed (WAIS digit symbol coding (Wechsler, 2008)). Processing speed predicts mortality (Swan *et al*., 1995) and is appealing because of its ease of measurement. Results were unchanged (Fig. S1, Supporting information).

To evaluate robustness of the results reported here, we conducted sensitivity analyses using two alternative algorithms to index biological aging. The Biological Age algorithm was originally developed using the US Centers for Disease Control and Prevention National Health and Nutrition Surveys (Levine, 2013). The Age‐related Homeostatic Dysregulation algorithm was originally developed using the Women's Health and Aging, Baltimore Longitudinal Study, and InCHIANTI cohorts (Cohen *et al*., 2013). Each algorithm combines data on multiple biomarkers, but from a single cross‐section of biomarker data. We applied these algorithms to biomarker data collected when Study members were aged 38 years. Study members with faster Pace of Aging were measured as having older Biological Age (*r* = 0.38, *P* < 0.001) (Belsky *et al*., 2015a). They also showed greater age‐related homeostatic dysregulation (*r* = 0.58, *P* < 0.001). Personal‐history characteristics showed similar patterns of association with these two cross‐sectional indexes as they did with the longitudinal Pace of Aging (Figs S2–S7, Supporting information).

## Discussion

Therapies to extend healthspan are poised to move from laboratory‐based model systems to trials in humans (de Cabo *et al*., 2014; Longo *et al*., 2015). Such human translation faces hurdles, including the substantial heterogeneity of human aging. To advance understanding of this heterogeneity, we studied how six personal‐history characteristics with documented relationships to later‐life morbidity and mortality predicted the pace of biological aging. We studied the pace of aging in young‐adult humans (aged 26–38 years) because therapies will need to be delivered by midlife in order to prevent onset of age‐related disease. Our principle finding is that young adults who show signs of rapid biological aging are characterized by personal‐history risks. Measuring future trial participants’ personal‐history characteristics will be important to designing rigorous tests of age‐delaying therapies.

We measured biological aging as the coordinated decline in integrity of multiple organ systems. We quantified the rate of this decline, the ‘Pace of Aging’ from repeated measurements of a panel of 18 biomarkers assayed when Study members were aged 26, 32, and 38 years (Belsky *et al*., 2015a). We measured personal histories, background characteristics hypothesized to influence biological aging, from archival data collected prospectively beginning during Study members’ childhoods. Study members’ familial longevity, childhood social class, childhood adverse experiences, and childhood health, intelligence, and self‐control were all related to their adulthood Pace of Aging. The relationship between personal‐history risks (e.g., adverse childhood experiences, low IQ) and biological aging was cumulative; more risks predicted faster Pace of Aging. This pattern of results was replicated when personal‐history risks were ascertained using quick, inexpensive contemporaneous measures, including adult Study members’ retrospective reports. The relationship between cumulative risk and faster Pace of Aging was similar in the subset of individuals with recent health system contact, who presumably better represent the population eligible to enroll in healthspan‐extension trials.

A primary implication of these findings is that healthspan‐extension trials can measure personal histories as one method to quantify heterogeneity in aging among potential participants. Measured personal histories may be useful to trials in at least four ways. First, enrolling participants with high personal‐history risk helps to ensure a trial includes participants who represent the population segment most in need of therapy. This is because individuals with high levels of personal‐history risk appear to be aging more rapidly and therefore will more urgently require healthspan‐extending interventions. Second, personal‐history information can inform evaluation of trial protocols. For example, personal‐history risks could be evaluated as predictors of adherence. Third, personal‐history characteristics, as sources of etiologic heterogeneity in aging, represent potential sources of variation in treatment response. Therefore, these characteristics may be useful to preregister as treatment effect modifiers. In other branches of medicine, personal‐history characteristics have been found to predict treatment response (Nanni *et al*., 2012). Fourth, personal histories can be ascertained cheaply and quickly. For example, our contemporaneous assessments (Table S1, Supporting information) required <3 min of the participants’ time.

If healthspan‐extension trials do adopt personal‐history assessments, they will need new team members. To date, interdisciplinarity in geroscience typically means biologists working with physicians. That collaboration is essential. Our findings argue that behavioral scientists too have a place in the design and conduct of healthspan‐extension trials. Tackling the challenge of translating healthspan‐extension therapies from worms, flies, and mice to free‐living humans will require geroscience teams with behavioral expertise (Staudinger, 2015).

A secondary implication is that individuals with high personal‐history risk may be well represented in populations seeking health care, and therefore should be available to enroll in trials. In the Dunedin cohort, individuals with recent prescription fills, and even those with recent hospital admissions showed similar or even elevated personal‐history risks relative to the cohort overall. An important question for future research is whether these high‐risk individuals will be willing to volunteer for trials and able to adhere to protocols. Midlife patients seeking care in medical centers could be surveyed on personal‐history characteristics (and willingness to volunteer) to create a subject pool for healthspan‐extension trials.

Our findings also have implications for etiological theories relating early‐life risk exposures to later‐life morbidity and mortality. Life course epidemiology and the study of developmental origins of health and disease document the link between early‐life adversity and later‐life disease and death (Ben‐Shlomo & Kuh, 2002; Gluckman & Hanson, 2004). Stress biology theory predicts this link is mediated by a process of ‘biological embedding’ (Danese & McEwen, 2012). Here, we provide evidence consistent with a hypothesis that biological embedding occurs through an acceleration of the aging process during the third and fourth decades of life. Intervention to slow aging may provide a path to mitigate damage caused by early adversity.

We acknowledge limitations. First, we studied a single New Zealand birth cohort that lacked ethnic minority representation. Replications are needed. Second, follow‐up was right censored at the fifth decade of life. Study members aging slowly during their third and fourth decades of life might experience acceleration subsequently. Similarly, more rapidly aging Study members might experience a slower Pace of Aging in later years. Analysis in cohorts of older individuals is needed. Third, our analysis concerned only risks accruing through childhood. We selected personal‐history characteristics from early life to establish temporal precedence of personal histories to Pace of Aging measured during Study members’ 20s and 30s. However, adolescent and young‐adult lifestyles and health behaviors likely influence aging (Crimmins *et al*., 2009) and should also be measured as sources of heterogeneity in clinical trials. Our analysis also omitted some early‐life exposures. Low birthweight and related perinatal risks predict late‐life disease and mortality (Barker *et al*., 2002). Should records of perinatal exposures be available to trials of therapies to slow aging, they may prove useful. Fourth, our retrospective battery lacked recall of childhood health. Retrospective childhood health assessments are used in social surveys of older adults, and predict aging outcomes in these samples (Blackwell *et al*. 2001).

Our measurement of accelerated aging is necessarily imperfect. Methods are still under development to quantify biological aging. The one we used, Pace of Aging, is distinct from many others in that it is based on observations of change occurring over time within individuals. In contrast, most other methods are based on comparisons of chronologically older individuals to younger ones. In acknowledgment of this difference, we repeated our analysis using two such indexes, the “Biological Age” (Levine, 2013), and an aging‐related homeostatic dysregulation index (Cohen *et al*., 2013), and obtained similar results. Pace of Aging predicts many signs of foreshortened healthspan: Faster Pace of Aging is related to poorer performance on tests of strength, balance, and motor coordination, signs of early‐onset cognitive decline, and aged facial appearance already by midlife (Belsky *et al*., 2015a). Biological Age and age‐related homeostatic dysregulation predict mortality (Levine & Crimmins, 2014; Li *et al*., 2015). Replication of findings with these indexes argues that our results are not sensitive to the specific method used to quantify biological aging. Nevertheless, further demonstrations with alternative methods of quantification can strengthen confidence in these findings.

In sum, this article links together two separate streams of research. One stream addresses early‐life exposures that shorten healthspan (Gluckman & Hanson, 2004; Epel, 2009; Danese & McEwen, 2012). The other, geroscience, seeks to develop interventions that modify aging biology to lengthen healthspan (Burch *et al*., 2014; Kennedy *et al*., 2014; Ravussin *et al*., 2015). Our analysis joins these streams at the life course stage when they intersect: middle life. Our results show this linkage is sound, and suggest that measuring early‐life personal histories of participants can benefit healthspan‐extension trials.

## Methods

### Sample

Participants are members of the Dunedin Study, a longitudinal investigation of health and behavior in a representative birth cohort. Study members (*N* = 1037; 91% of eligible births; 52% male) were all individuals born between April 1972 and March 1973 in Dunedin, New Zealand (NZ), who were eligible based on residence in the province and who participated in the first assessment at age 3 (Poulton *et al*., 2015). The cohort represents the full range of socioeconomic status on NZ's South Island and matches the NZ National Health and Nutrition Survey on key health indicators (e.g., BMI, smoking, GP visits) (Poulton *et al*., 2015). Cohort members are primarily white; fewer than 7% self‐identify as having partial non‐Caucasian ancestry, matching the South Island. Assessments were carried out at birth and ages 3, 5, 7, 9, 11, 13, 15, 18, 21, 26, 32, and, most recently, 38 years, when 95% of the 1007 Study members still alive took part. At each assessment, each Study member is brought to the research unit for a full day of interviews and examinations. The Otago Ethics Committee approved each phase of the study and informed consent was obtained from all Study members.

In addition to studying the full cohort, we used electronic medical record data to identify two subsamples within the cohort who had recent contact with the healthcare system. The first subsample comprises cohort members with recent pharmaceutical prescriptions. This subsample was identified via record linkage with the Pharmaceutical Management Agency (PHARMAC) database. PHARMAC is the New Zealand Crown agency that selects and purchases medicines that are subsidized for use in the community (all common medications are on the list). The database represents a record of requests from pharmacists for payment of subsidies associated with prescriptions linked to the National Health Index (NHI) number, a unique identifier assigned to every person who accesses health‐related support in New Zealand. The second subsample comprises cohort members with recent hospital admissions. This subsample was identified via record linkage with the New Zealand Ministry of Health. The database contains information about admission events to nationwide hospitals and is linked to the NHI number. Both searches were conducted at the end of the last assessment (age 38 years), and here, we report data about the time period since the previous assessment at age 32 years.

### Measuring the Pace of Aging

As described previously (Belsky *et al*., 2015a), we measured Pace of Aging in *n* = 954 Dunedin Study members with repeated assessments of a panel of 18 biomarkers taken at ages 26, 32, and 38 tears. The biomarkers were as follows: apolipoprotein B100/A1 ratio, blood pressure (mean arterial pressure), body mass index (BMI) and waist–hip ratio, C‐reactive protein and white blood cell count, cardiorespiratory fitness (VO_2_Max), creatinine clearance, forced expiratory volume in one‐second (FEV_1_) and forced vital capacity ratio (FEV_1_/FVC), glycated hemoglobin, high‐density lipoprotein (HDL), lipoprotein(a), leukocyte telomere length (LTL), periodontal disease, total cholesterol, triglycerides, and urea nitrogen. For each biomarker, we calculated the Study member's personal rate of change using mixed‐effects growth models. We combined these rates of change into a single index scaled in years of physiological change occurring per one chronological year. The average Study member had Pace of Aging equal to 1 year of physiological change per one chronological year. The fastest‐aging Study members experienced more than twice that rate of physiological change. The slowest‐aging Study members experienced almost no change at all. The resulting Pace of Aging measure took on a normal distribution with M = 1, SD = 0.38.

#### Cross‐sectional biological age at age 38 years

As described previously (Belsky *et al*., 2015a), we calculated each Dunedin Study member's Biological Age at age 38 years using the Klemera–Doubal equation (Klemera & Doubal, 2006) and parameters estimated from the NHANES‐III dataset (Levine, 2013) for ten biomarkers (seven of which overlapped with Pace of Aging): glycated hemoglobin, forced expiratory volume in one‐second (FEV_1_), blood pressure (systolic), total cholesterol, C‐reactive protein, creatinine, urea nitrogen, albumin, alkaline phosphatase, and cytomegalovirus IgG. Biological Age took on a normal distribution, ranging from 28 to 61 years (M = 38 years, SD = 3.23).

#### Cross‐sectional age‐related homeostatic dysregulation at age 38 years

We measured age‐related homeostatic dysregulation by applying the biomarker Mahalanobis distance method described by Cohen and colleagues (Cohen *et al*., 2013; Li *et al*., 2015) to Study members’ age‐38 biomarker values. Biomarker Mahalanobis distance measures how aberrant an individual's physiology is relative to a reference norm (Cohen *et al*., 2013). Cohen and colleagues used chronologically young individuals to form this reference norm for their calculations (Li *et al*., 2015). They interpreted biomarker Mahalanobis distance from the reference as an indicator of age‐related homeostatic dysregulation, a sign of biological aging. We formed our reference from the Dunedin Study members’ biomarker values at age 26 years, the youngest age at which the biomarkers were measured. We then calculated Mahalanobis distance based on age‐38 values of the 18 biomarkers included in Pace of Aging calculation. Thus, a Study member's biomarker Mahalanobis distance quantifies decline in homeostatic regulation relative to the cohort's age‐26 norm. Distances were log‐transformed and standardized to have M = 1, SD = 0 for analysis.

### Measuring personal histories

We assessed six personal‐history characteristics hypothesized to relate to biological aging using archival data prospectively collected beginning when Study members were children. The six personal‐history characteristics were familial longevity, childhood social class, adverse childhood experiences (ACEs), and childhood physical health, intelligence, and self‐control. We also measured personal histories from contemporaneous measures including retrospective reports collected from Study members when they were adults. Details on personal‐history measurements are reported in the Supplementary Information.

## Funding

The Dunedin Multidisciplinary Health and Development Research Unit is supported by the New Zealand Health Research Council and New Zealand Ministry of Business, Innovation, and Employment (MBIE). This research received support from US National Institute of Aging grants R01AG032282, R01AG048895, and 1R01AG049789, UK Medical Research Council grant MR/P005918/1, and UK ESRC grant ES/M010309/1. Additional support was provided by P30AG028716 and P30AG034424 and by the Jacobs Foundation. DWB is supported by an Early‐Career Research Fellowship from the Jacobs Foundation.

## Author contributions

DWB, AC, and TEM conceived the study, carried out analyses, and wrote the manuscript. AC, SR, RP, and TEM collected the data. HJC, WEK, SR, and RP reviewed manuscript drafts and provided critical feedback.

## Conflict of interest

None declared.

## Supporting information


**Fig. S1** Proportions of members with slow, average, and fast Pace of Aging classified as high‐risk on 0, 1, or 2‐or‐more family and childhood characteristics based on assessments conducted in adulthood.
**Fig. S2** Family and childhood characteristics are associated with Study members’ Biological Age measured at chronological age 38 years.
**Fig. S3** Cumulative prospectively‐assessed personal‐history risks in Study members with Biological Ages younger than 35, between 35 and 41, and older than 41.
**Fig. S4** Proportions of members with Biological Age <35, 35–41, and >41 classified as high‐risk on 0, 1, or 2‐or‐more family and childhood characteristics based on contemporaneous assessments conducted in adulthood.
**Fig. S5** Family and childhood characteristics are associated with Study members’ age‐related homeostatic dysregulation measured at chronological age 38 years.
**Fig. S6** Cumulative prospectively‐assessed personal‐history risks in Study members with low, average, and high levels of age‐related homeostatic dysregulation.
**Fig. S7** Proportions of members with low, average, and high levels of agerelated homeostatic dysregulation classified as high‐risk on 0, 1, or 2‐or‐more family and childhood characteristics based on contemporaneous assessments conducted in adulthood.
**Table S1** Adult interview to ascertain personal history risks for accelerated aging.
