# Supplementary material for: Impact of early personal‐history characteristics on the Pace of Aging: implications for clinical trials of therapies to slow aging and extend healthspan
Source: Aging Cell. 2017 Apr 12;16(4):644–51. doi: 10.1111/acel.12591 (PMC5506399; doi:10.1111/acel.12591)
Supplement: Supplementary file 1 — Fig. S1 Proportions of members with slow, average, and fast Pace of Aging classified as high‐risk on 0, 1, or 2‐or‐more family and childhood characteristics based on assessments conducted in adulthood. Fig. S2 Family and childhood characteristics are associated with Study members’ Biological Age measured at chronological age 38 years. Fig. S3 Cumulative prospectively‐assessed personal‐history risks in Study members with Biological Ages younger than 35, between 35 and 41, and older than 41. Fig. S4 Proportions of members with Biological Age <35, 35–41, and >41 classified as high‐risk on 0, 1, or 2‐or‐more family and childhood characteristics based on contemporaneous assessments conducted in adulthood. Fig. S5 Family and childhood characteristics are associated with Study members’ age‐related homeostatic dysregulation measured at chronological age 38 years. Fig. S6 Cumulative prospectively‐assessed personal‐history risks in Study members with low, average, and high levels of age‐related homeostatic dysregulation. Fig. S7 Proportions of members with low, average, and high levels of agerelated homeostatic dysregulation classified as high‐risk on 0, 1, or 2‐or‐more family and childhood characteristics based on contemporaneous assessments conducted in adulthood. Table S1 Adult interview to ascertain personal history risks for accelerated aging. [file ACEL-16-644-s001.pdf]

SUPPLEMENTARY INFORMATION

Measurement of Personal History Characteristics

Familial longevity. As part of the Dunedin Family Health History Study *(Milne et al. 2009)*, carried out when Study members were aged 32 years, we interviewed parents of Study members about their family history, including the current age or age at death for each of the Study member's biological grandparents. Information was collected on 3,548 biological grandparents (51% female), of whom 77% had died by the time of the interview. The median (inter-quartile range) of grandparent age was 78 (70-84) for grandmothers and 73 (63-81) for grandfathers.

We analyzed familial longevity in two ways: As the age of the longest-lived grandparent in a Study member's family (M=84, SD=7 years), and as a dichotomous indicator of a "short-lived" family, defined as no grandparent having lived to age 80 years (24% of Study members). We selected 80 years as the threshold because this is approximately the life expectancy for New Zealanders of the grandparents' generation who survived to childbearing age (Statistics New Zealand, Accessed 2/1/2016).

Social class origins. As described previously *(Poulton et al. 2002)*, Study members' socioeconomic status during childhood was defined as the average of the highest occupational status level of either parent across study assessments from the Study member's birth through 15 years (1=unskilled laborer; 6=professional), on New Zealand's occupational rating of the 1970's *(Elley & Irving 1976)*.

We analyzed Study members' social class origins as a continuous index (M=3.75, SD=1.14) and as a dichotomous indicator of low social class origins (a score of 2 or lower, 21% of Study members).

Adverse childhood experiences (ACEs). ACEs are any potentially damaging traumatic or stressful conditions to which an individual was exposed during the first 15 years of life. Our measure of ACEs corresponds to the 10 categories of childhood adversity introduced by the CDC Adverse Childhood Experiences Study *(Felitti et al. 1998)* (http://www.cdc.gov/violenceprevention/acestudy/prevalence.html): Five types of child harm

(including physical abuse, sexual abuse, emotional abuse, physical neglect and emotional neglect) and five types of household dysfunction (including, household substance abuse, household mental illness, incarceration of a family member, partner violence, and parental loss).

Prospective ACE counts were generated from archival Dunedin Study records gathered during 7 biennial assessments carried out from ages 3 to 15 years. The records include: social service contacts; structured notes from assessment staff who interviewed Study children and their parents; structured notes from pediatricians and psychometricians who observed mother-child interactions at the research unit; structured notes from nurses who recorded conditions witnessed at home visits; and notes of concern from teachers who were surveyed about the Study children's behavior and performance. Separately, parental criminality was surveyed via postal questionnaire to the parents. Archival Study data were reviewed in 2015 by four independent raters who were trained on the CDC definitions of ACEs. Individual ACEs were agreed upon by at least three of the four raters 80% of time. The sole exception was emotional neglect where half the cases were identified by only two raters. Agreement across the full ACE count between the four raters ranged from kappa = 0.76 to 0.82, with an average inter-rater agreement kappa of 0.79.

We analyzed prospectively collected ACEs in two ways: As a continuous index (M=1.05, SD=1.19) and, following the CDC ACE study *(Felitti et al. 1998)*, as a dichotomous indicator of having four or more ACEs (7% of Study members).

Childhood health. As described previously *(Belsky, Caspi, Israel, et al. 2015)*, we measured childhood health from exams (balance, blood pressure, anthropometry, lung function testing by spirometry), nurse ratings, and clinical interviews with parents at assessments spanning ages 3-11 years. To calculate the childhood health measure, assessments were standardized to have mean=0 SD=1 within age and sex specific groups. Cross-age scores for

each measure were then computed by averaging standardized scores across measurement ages. The final childhood health score was calculated by taking the natural log of the average score across all measures, resulting in a normally distributed childhood health index.

We analyzed childhood health as a continuous index (M=0, SD=1) and as a dichotomous indicator of poor childhood health, defined as 1 SD below the mean (childhood health score <-1, 15% of Study members).

Childhood intelligence (IQ). As previously described *(Moffitt et al. 2011)*, the Wechsler Intelligence Scale for Children – Revised (WISC-R) *(Wechsler 2003)* was individually administered at ages 7, 9, 11, and 13 years. IQ scores for the four ages were averaged and standardized.

We analyzed childhood IQ as a continuous index (M=100, SD=15) an as a dichotomous indicator of low IQ, defined as 1 SD below the mean (IQ<85, 15% of Study members).

Childhood self-control. As previously described *(Moffitt et al. 2011)*, children's self-control during their first decade of life was measured using nine measures of self-control: observational ratings of children's lack of control (ages 3 and 5 years) and parent, teacher, and self-reports of hyperactivity, lack of persistence, inattention, impulsive aggression and impulsivity (ages 5, 7, 9, and 11 years). The nine measures were positively and significantly correlated. Based on principal components analysis, the standardized measures were averaged into a single composite comprising multiple ages and informants, with strong internal reliability $\alpha = 0.86$.

We analyzed childhood self-control as a continuous index (M=0, SD=1) and as a dichotomous indicator of low self-control, defined as 1 SD below the mean (self-control<-1, 15% of Study members). Correlations among personal-history characteristics are shown below.

	(1)	(2)	(3)	(4)	(5)
(1) Age of Longest-lived Grandparent	--				
(2) Childhood Social Class	0.30	--			
(3) Adverse Childhood Experiences	-0.21	-0.29	--		
(4) Childhood Physical Health	0.07	0.15	-0.09	--	
(5) Childhood IQ	0.14	0.42	-0.22	0.20	--
(6) Childhood Self-control	0.19	0.25	-0.30	0.15	0.45

Personal History Assessments Implemented in Adulthood

Clinical trials of interventions to slow aging that enroll adults may not have access to prospective data on childhood risks. Therefore, we also measured Study members' risks for accelerated aging using data that could be collected at the time of enrollment into a trial.

Familial longevity. Familial longevity was assessed using the methodology described above. We identified individuals with short-lived families as those whose grandparents had all died before the age of 80 years (24% of Study members).

Retrospective assessment of social class origins. Adult Study members were asked what their parents had done for a living during the Study member's childhood. Reported occupations were coded on a 1-6 scale according to the New Zealand Socioeconomic Index (1=unskilled laborer; 6=professional) *(Milne et al. 2013)*. We identified Study members with low social class origins as those who reported their parents to have had an occupation that was coded on the index as low-status (score of 2 of lower, 23% of Study members).

Retrospective assessment of Adverse Childhood Experiences (ACEs). Study members were interviewed about each of the 10 categories of childhood adversity in the CDC ACE Study *(Felitti et al. 1998)*. We interviewed about physical, sexual and emotional abuse, physical neglect and emotional neglect via the Childhood Trauma Questionnaire (CTQ) *(Bernstein & Fink 1998)*. The validity of the instrument has been previously demonstrated in clinical and community samples *(Bernstein et al. 1997; Scher et al. 2001)*. We used the score classification evaluated and recommended by the CTQ manual and considered a specific category of harm present if the Study member had a moderate to severe score. We interviewed about substance abuse, mental illness, and incarceration via the Family History Screen for assessing family history of mental disorders *(Milne et al. 2009)*. We assessed exposure to partner violence by asking Study participants, ""Did you ever see or hear about your mother/father being hit or hurt by your father/ mother/ stepfather/ stepmother"? We assessed parental loss by asking Study participants if they had ever lost a parent. We identified Study members with a high burden of retrospectively reported ACEs as those reporting four or more ACEs, following the CDC ACE study *(Felitti et al. 1998)* (15% of Study members).

Educational attainment. As a proxy for childhood IQ, we measured Study members' educational attainment. For the 1972-73 birth cohort we studied, compulsory education ended at age 15 years, at which point students could elect to sit for a School Leaving Certificate exam. 16% of our sample obtained no educational credential. 15% obtained the School Leaving Certificate but did not progress further. 41% completed 6th form or Bursary Certificates (roughly equivalent to a full high school diploma in the United States). 28% completed a university degree. Following established practice for analysis of New Zealand educational data *(Fergusson et al. 2005)*, we identified Study members with low levels of education as those who had not obtained any educational credential (16% of Study members).

Processing Speed. The digit-symbol coding task of the Wechsler Adult Intelligence Scale *(Wechsler 2008)* is a nonverbal test of processing speed. A key that pairs symbols and numbers is presented. The test requires filling in rows containing blank squares (each with a randomly assigned number above it) using the key. The test has a time limit of two minutes. We coded low processing speed as providing the correct code for fewer than a threshold number of the 135 symbols on the test. We set that threshold at 1 SD below the age-norm (approximately 50 for midlife samples according to the Wechsler Adult Intelligence Scale Scoring Manual, p. 211 *(Wechsler 2008)*).

Conscientiousness. As a proxy for childhood self-control, we measured adult Study members' level of Conscientiousness, a personality trait that describes self-discipline, planfulness, and dependability. As previously described *(Israel et al. 2014)*, Conscientiousness was assessed at the age-38 exam by the nurse who guided the Study member through the physical exam and, afterwards, completed a brief personality inventory describing the Study member. Conscientiousness was assessed by 5 bipolar items (e.g., quitting…..persevering; undependable….dependable) rated on a 7-point scale, with 4 as the scale midpoint. We counted up the number of items on which the nurse rated the study member as having the high conscientiousness characteristic (scores of 5 or greater). We identified Study members with low conscientiousness as those having only one or zero high conscientiousness ratings (12% of Study members).

Supplementary Table 1. Adult interview to ascertain personal history risks for accelerated aging.

Personal History Characteristic	Measurement	Risk value
Familial Longevity	"Have any of your grandparents lived to age 80 years or above?"	1 point for an answer of "No"
Childhood Social Class	"What was your mother's occupation when you were growing up?"<br>"What was your father's occupation when you were growing up?"<br>(The Dunedin Study asked specifically about parental occupations at age 15 years)<br><br>Occupational prestige is coded by matching the reported occupations to a coding scheme, e.g. the International Standard Classification of Occupations (ISCO) (14). (We used a version customized for New Zealand (8).) Classification is made based on the highest-prestige occupation of either parent.	1 point for having a low occupational prestige score. Using the ISCO classification, a low social-class score is below 30 ("Food service counter attendant" has an occupational prestige score of 25, "Child care workers and teachers' aides" have a score of 26).
Adverse Childhood Experiences (ACEs)	US Centers for Disease Control and Prevention ACE Study scale (available in ref (4), p. 248)	1 point for a score of 4 or greater
Childhood Intelligence	"What is the highest level of schooling you have completed?"	1 point for having a low level of educational attainment. (We used not having any educational credential.)
	2-minute nonverbal digit-symbol coding task from the Wechsler Adult Intelligence Scale, Fourth Edition (WAIS-IV) (13).	1 point for scoring more than 1 SD below the age-specific test-norm (a score of 50 or below for midlife samples)
Childhood Self Control	Nurse (or other research worker) rating on 5-item conscientiousness scale (see below).	1 point for receiving below-average scores on at least 4 of the 5 items. (in the Dunedin cohort, a score of 4 or below was below average)

Scale to measure participant conscientiousness (To be completed by a research worker who has interacted with the participant. In the Dunedin Study, scales were completed by nurses who conducted physical exams.)

1<br>Impatient	2	3	4	5	6	7<br>Patient
1<br>Careless	2	3	4	5	6	7<br>Fussy/tidy
1<br>Undependable	2	3	4	5	6	7<br>Responsible
1<br>Unexacting	2	3	4	5	6	7<br>Exacting
1<br>Quitting	2	3	4	5	6	7<br>Persevering

REFERENCES

Atzmon G, Schechter C, Greiner W, Davidson D, Rennert G & Barzilai N (2004) Clinical phenotype of families with longevity. *J. Am. Geriatr. Soc.* 52, 274–277.

Barker DJP, Eriksson JG, Forsén T & Osmond C (2002) Fetal origins of adult disease: strength of effects and biological basis. *Int. J. Epidemiol.* 31, 1235–1239.

Barnett K, Mercer SW, Norbury M, Watt G, Wyke S & Guthrie B (2012) Epidemiology of multimorbidity and implications for health care, research, and medical education: a cross-sectional study. *Lancet* 380, 37–43.

Belsky DW, Caspi A, Houts R, Cohen HJ, Corcoran DL, Danese A, Harrington H, Israel S, Levine ME, Schaefer JD, Sugden K, Williams B, Yashin AI, Poulton R & Moffitt TE (2015) Quantification of biological aging in young adults. *Proc. Natl. Acad. Sci. U. S. A.* 112, E4104–4110.

Belsky DW, Caspi A, Israel S, Blumenthal JA, Poulton R & Moffitt TE (2015) Cardiorespiratory fitness and cognitive function in midlife: Neuroprotection or neuroselection? *Ann. Neurol.* 77, 607–617.

Bernstein DP, Ahluvalia T, Pogge D & Handelsman L (1997) Validity of the Childhood Trauma Questionnaire in an adolescent psychiatric population. *J. Am. Acad. Child Adolesc. Psychiatry* 36, 340–348.

Bernstein DP & Fink L (1998) *Childhood trauma questionnaire: A retrospective self-report: Manual*, Psychological Corporation.

Blackwell DL, Hayward MD & Crimmins EM (2001) Does childhood health affect chronic morbidity in later life? *Soc. Sci. Med. 1982* 52, 1269–1284.

Burch JB, Augustine AD, Frieden LA, Hadley E, Howcroft TK, Johnson R, Khalsa PS, Kohanski RA, Li XL, Macchiarini F, Niederehe G, Oh YS, Pawlyk AC, Rodriguez H, Rowland JH, Shen GL, Sierra F & Wise BC (2014) Advances in geroscience: impact on healthspan and chronic disease. *J. Gerontol. A. Biol. Sci. Med. Sci.* 69 Suppl 1, S1–3.

De Cabo R, Carmona-Gutierrez D, Bernier M, Hall MN & Madeo F (2014) The Search for Antiaging Interventions: From Elixirs to Fasting Regimens. *Cell* 157, 1515–1526.

Calvin CM, Deary IJ, Fenton C, Roberts BA, Der G, Leckenby N & Batty GD (2011) Intelligence in youth and all-cause-mortality: systematic review with meta-analysis. *Int. J. Epidemiol.* 40, 626–644.

Case A, Fertig A & Paxson C (2005) The lasting impact of childhood health and circumstance. *J. Health Econ.* 24, 365–389.

Cohen AA, Li Q, Milot E, Leroux M, Faucher S, Morissette-Thomas V, Legault V, Fried LP & Ferrucci L (2015) Statistical distance as a measure of physiological dysregulation is largely robust to variation in its biomarker composition. *PloS One* 10, e0122541.

Cohen AA, Milot E, Yong J, Seplaki CL, Fülöp T, Bandeen-Roche K & Fried LP (2013) A novel statistical approach shows evidence for multi-system physiological dysregulation during aging. *Mech. Ageing Dev.* 134, 110–117.

Danese A & McEwen BS (2012) Adverse childhood experiences, allostasis, allostatic load, and age-related disease. *Physiol. Behav.* 106, 29–39.

Elley WB & Irving JC (1976) Revised socioeconomic index for New-Zealand. *N. Z. J. Educ. Stud.* 11, 25–36.

Epel ES (2009) Telomeres in a life-span perspective a new "psychobiomarker"? *Curr. Dir. Psychol. Sci.* 18, 6–10.

Evans GW, Li D & Whipple SS (2013) Cumulative risk and child development. *Psychol. Bull.* 139, 1342–1396.

Felitti VJ, Anda RF, Nordenberg D, Williamson DF, Spitz AM, Edwards V, Koss MP & Marks JS (1998) Relationship of childhood abuse and household dysfunction to many of the leading causes of death in adults. The Adverse Childhood Experiences (ACE) Study. *Am. J. Prev. Med.* 14, 245–258.

Fergusson DM, John Horwood L & Ridder EM (2005) Show me the child at seven: the consequences of conduct problems in childhood for psychosocial functioning in adulthood. *J. Child Psychol. Psychiatry* 46, 837–849.

Friedman HS, Tucker JS, Tomlinson-Keasey C, Schwartz JE, Wingard DL & Criqui MH (1993) Does childhood personality predict longevity? *J. Pers. Soc. Psychol.* 65, 176–185.

Gavrilov LA & Gavrilova NS (2004) Early-life programming of aging and longevity: the idea of high initial damage load (the HIDL hypothesis). *Ann. N. Y. Acad. Sci.* 1019, 496–501.

Gillman MW (2005) Developmental origins of health and disease. *N. Engl. J. Med.* 353, 1848–1850.

Gluckman PD & Hanson MA (2004) Living with the past: evolution, development, and patterns of disease. *Science* 305, 1733–1736.

Guarente L (2014) Aging research-where do we stand and where are we going? *Cell* 159, 15–19.

Harper S (2014) Economic and social implications of aging societies. *Science* 346, 587–591.

Hayward MD & Gorman BK (2004) The long arm of childhood: the influence of early-life social conditions on men's mortality. *Demography* 41, 87–107.

Israel S, Moffitt TE, Belsky DW, Hancox RJ, Poulton R, Roberts B, Murray W & Caspi A (2014) Translating personality psychology to help personalize preventive medicine for young adult patients. *J. Pers. Soc. Psychol.* 106, 484–498.

Kaeberlein M (2013) Longevity and aging. *F1000Prime Rep.* 5. Available at: http://f1000.com/prime/reports/b/5/5/ [Accessed October 9, 2013].

Kennedy BK, Berger SL, Brunet A, Campisi J, Cuervo AM, Epel ES, Franceschi C, Lithgow GJ, Morimoto RI, Pessin JE, Rando TA, Richardson A, Schadt EE, Wyss-Coray T & Sierra F (2014) Geroscience: linking aging to chronic disease. *Cell* 159, 709–713.

Kirkland JL (2013) Translating advances from the basic biology of aging into clinical application. *Exp. Gerontol.* 48, 1–5.

Kirkwood TB & Austad SN (2000) Why do we age? *Nature* 408, 233–8.

Klemera P & Doubal S (2006) A new approach to the concept and computation of biological age. *Mech. Ageing Dev.* 127, 240–248.

Kuh D, Ben-Shlomo Y, Lynch J, Hallqvist J & Power C (2003) Life course epidemiology. *J. Epidemiol. Community Health* 57, 778–83.

Ledford H (2015) How to solve the world's biggest problems. *Nature* 525, 308–311.

Levine ME (2013) Modeling the rate of senescence: Can estimated biological age predict mortality more accurately than chronological age? *J. Gerontol. A. Biol. Sci. Med. Sci.* 68, 667–674.

Levine ME & Crimmins EM (2014) A comparison of methods for assessing mortality risk. *Am. J. Hum. Biol. Off. J. Hum. Biol. Counc.* 26, 768–776.

Li Q, Wang S, Milot E, Bergeron P, Ferrucci L, Fried LP & Cohen AA (2015) Homeostatic dysregulation proceeds in parallel in multiple physiological systems. *Aging Cell* 14, 1103–1112.

Longo VD, Antebi A, Bartke A, Barzilai N, Brown-Borg HM, Caruso C, Curiel TJ, de Cabo R, Franceschi C, Gems D, Ingram DK, Johnson TE, Kennedy BK, Kenyon C, Klein S, Kopchick JJ, Lepperdinger G, Madeo F, Mirisola MG, Mitchell JR, Passarino G, Rudolph KL, Sedivy JM, Shadel GS, Sinclair DA, Spindler SR, Suh Y, Vijg J, Vinciguerra M & Fontana L (2015) Interventions to Slow Aging in Humans: Are We Ready? *Aging Cell*.

López-Otín C, Blasco MA, Partridge L, Serrano M & Kroemer G (2013) The hallmarks of aging. *Cell* 153, 1194–1217.

Milne BJ, Byun U & Lee A (2013) *New Zealand socio-economic index 2006.*, Wellington, NZ: Statistics New Zealand.

Milne BJ, Caspi A, Harrington H, Poulton R, Rutter M & Moffitt TE (2009) Predictive Value of Family History on Severity of Illness The Case for Depression, Anxiety, Alcohol Dependence, and Drug Dependence. *Arch. Gen. Psychiatry* 66, 738–747.

Moffitt TE, Arseneault L, Belsky D, Dickson N, Hancox RJ, Harrington H, Houts R, Poulton R, Roberts BW, Ross S, Sears MR, Thomson WM & Caspi A (2011) A gradient of childhood self-control predicts health, wealth, and public safety. *Proc. Natl. Acad. Sci.* 108, 2693–8.

Moskalev A, Chernyagina E, Tsvetkov V, Fedintsev A, Shaposhnikov M, Krut'ko V, Zhavoronkov A & Kennedy BK (2016) Developing criteria for evaluation of geroprotectors as a key stage toward translation to the clinic. *Aging Cell*.

Nanni V, Uher R & Danese A (2012) Childhood Maltreatment Predicts Unfavorable Course of Illness and Treatment Outcome in Depression: A Meta-Analysis. *Am. J. Psychiatry* 169, 141–151.

Perls T & Terry D (2003) Understanding the determinants of exceptional longevity. *Ann. Intern. Med.* 139, 445–449.

Pitt JN & Kaeberlein M (2015) Why Is Aging Conserved and What Can We Do about It? *PLoS Biol.* 13, e1002131.

Poulton R, Caspi A, Milne BJ, Thomson WM, Taylor A, Sears MR & Moffitt TE (2002) Association between children's experience of socioeconomic disadvantage and adult health: a life-course study. *Lancet* 360, 1640–1645.

Poulton R, Moffitt TE & Silva PA (2015) The Dunedin Multidisciplinary Health and Development Study: overview of the first 40 years, with an eye to the future. *Soc. Psychiatry Psychiatr. Epidemiol.* 50, 679–693.

Power C, Kuh D & Morton S (2013) From developmental origins of adult disease to life course research on adult disease and aging: insights from birth cohort studies. *Annu. Rev. Public Health* 34, 7–28.

Ravussin E, Redman LM, Rochon J, Das SK, Fontana L, Kraus WE, Romashkan S, Williamson DA, Meydani SN, Villareal DT, Smith SR, Stein RI, Scott TM, Stewart TM, Saltzman E, Klein S, Bhapkar M, Martin CK, Gilhooly CH, Holloszy JO, Hadley EC, Roberts SB & CALERIE Study Group (2015) A 2-Year Randomized Controlled Trial of Human Caloric Restriction: Feasibility and Effects on Predictors of Health Span and Longevity. *J. Gerontol. A. Biol. Sci. Med. Sci.* 70, 1097–1104.

Scher CD, Stein MB, Asmundson GJ, McCreary DR & Forde DR (2001) The childhood trauma questionnaire in a community sample: psychometric properties and normative data. *J. Trauma. Stress* 14, 843–857.

Staudinger UM (2015) Towards Truly Interdisciplinary Research on Human Development. *Res. Hum. Dev.* 12, 335–341.

Swan GE, Carmelli D & LaRue A (1995) Performance on the Digit Symbol Substitution Test and 5-Year Mortality in the Western Collaborative Group Study. *Am. J. Epidemiol.* 141, 32–40.

Wechsler D (2008) *Wechsler Adult Intelligence Scale* 4th ed., San Antonio, TX: Pearson Assessment.

Wechsler D (2003) *Wechsler Intelligence Scale for Children* 4th (UK Version)., San Antonio, TX: Harcourt Assessment.

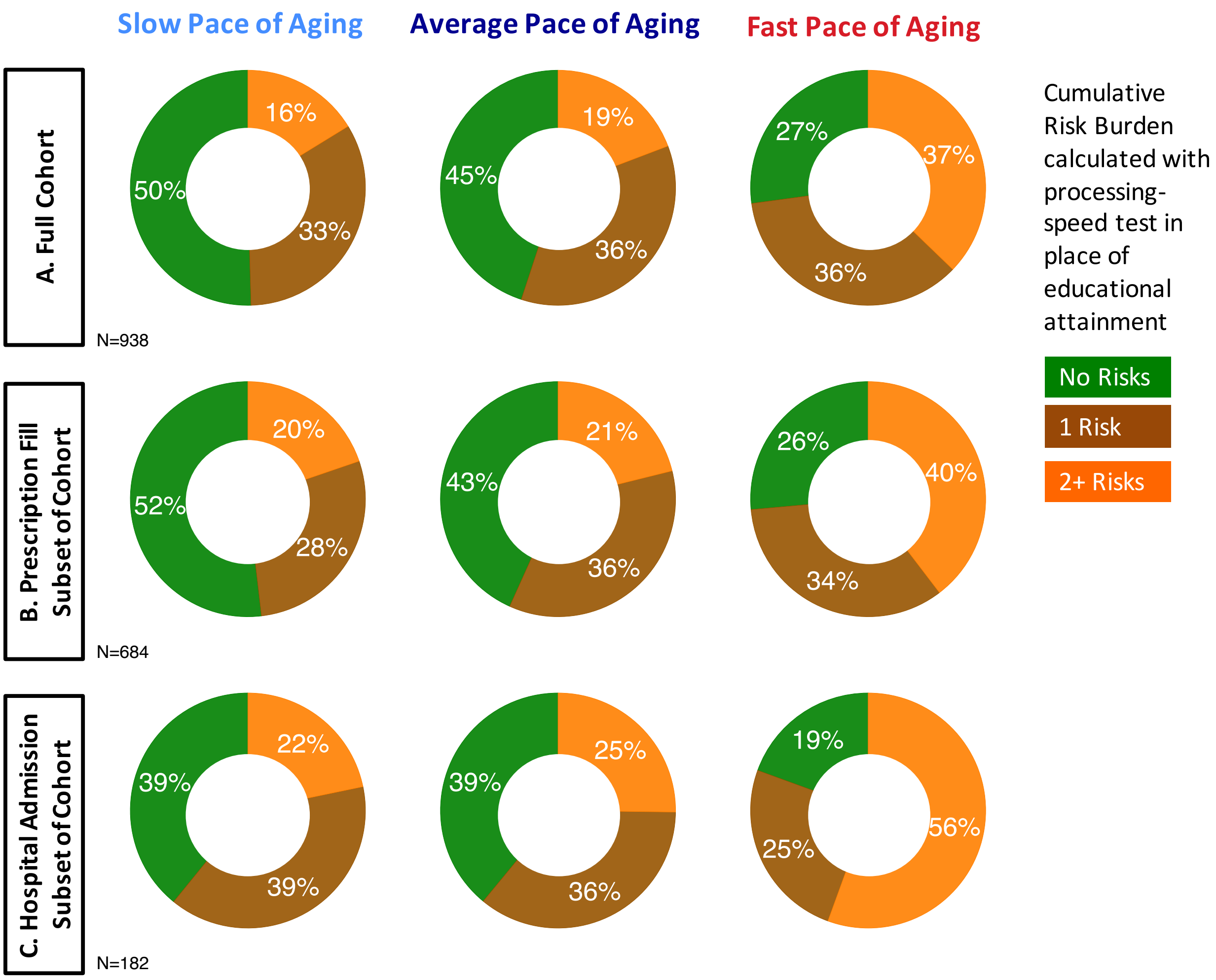

Supplemental Figure 1. Proportions of members with slow, average, and fast Pace of Aging classified as high-risk on 0, 1, or 2-or-more family and childhood characteristics based on assessments conducted in adulthood. Risk factors were having short-lived grandparents (no grandparent survived past age 80 years), retrospective report by the Study member that their parents held low-status occupations during the Study member's childhood, retrospective report of exposure to 4 or more adverse childhood experiences, having a low processing speed (digit-symbol coding) score, and being rated by an examining nurse as having low levels of the personality trait conscientiousness. **Panel A** graphs results for the full cohort. The pattern is the same as when risk was classified from assessments during childhood. Most slow-aging Study members were not classified as high-risk on any family or childhood characteristic. In contrast, nearly 40% of the fast-aging Study members were classified as high-risk on multiple family and childhood characteristics. Panels B and C repeat the analysis for subsamples of cohort members with recent contacts with the health care system and who may reflect the population most accessible to recruitment into clinical trials. **Panel B** graphs results for Study members with a recent prescription fill. **Panel C** graphs results for Study members with a recent hospital admission (excluding for pregnancy-related services).

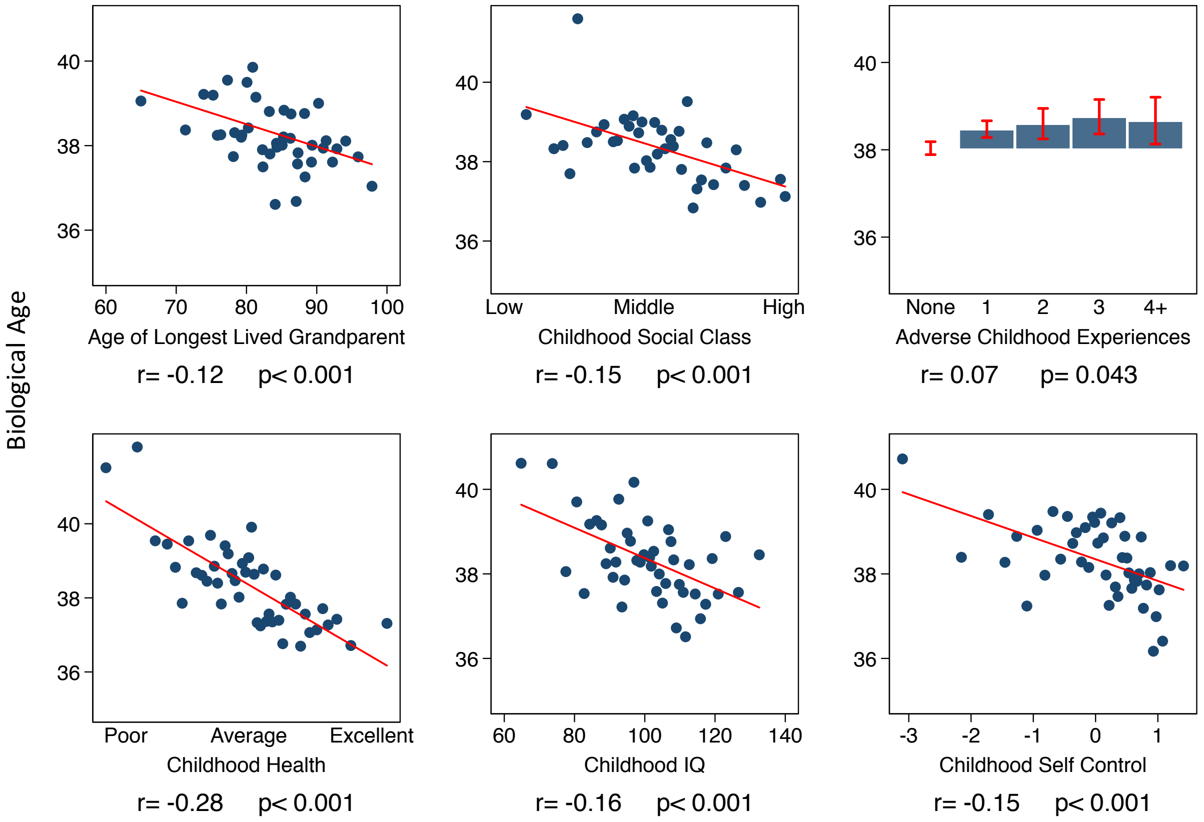

Supplemental Figure 2. Family and childhood characteristics are associated with Study members' Biological Age measured at chronological age 38 years. Figure cells graph associations between 6 family and childhood characteristics and Study members' Biological Age. As described previously *(Belsky, Caspi, Houts, et al. 2015; Levine 2013)*, Biological Age was measured from levels of 10 biomarkers using the Klemera-Doubal equation-based algorithm developed from the US Centers for Disease Control and Prevention NHANES database. Age of longest-lived grandparent was measured from reports by Study members' parents. Childhood social class, exposure to adverse childhood experiences, childhood health, childhood IQ, and childhood self-control were assessed using previously established methodology applied to archival Dunedin Study records including exams and testing, reports by parents and teachers, clinician ratings, administrative records, and direct observations. Figures show "binned" scatterplots in which each plotted point reflects average x- and y-coordinates for "bins" of approximately 20 Study members. Regression lines and effect-size estimates were estimated from the original, un-binned data.

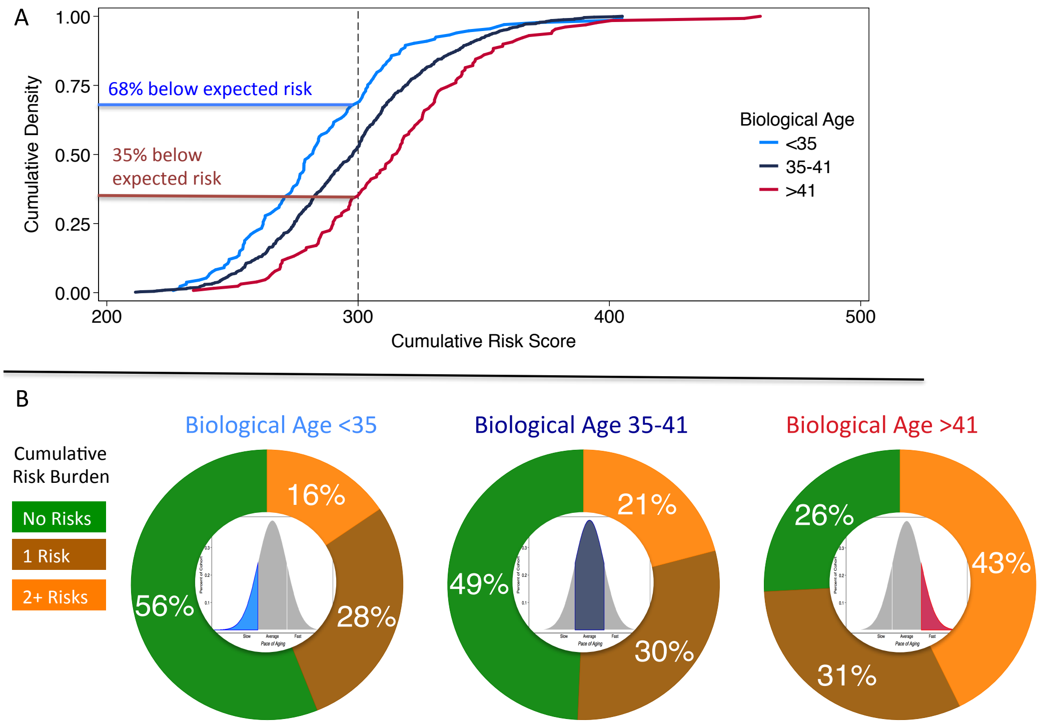

Supplemental Figure 3. Cumulative prospectively-assessed personal-history risks in Study members with Biological Ages younger than 35, between 35 and 41, and older than 41. Panel A graphs density plots of cumulative risk scores for Study members with Biological Age 1 SD or more younger than the cohort mean (<35 years), within 1 SD of the cohort mean (35-41 years), and >1 SD or more older than the cohort mean (>41 years). The cumulative risk score reflects total burden of risk across 6 personal history characteristics (grandparent longevity, family social class during childhood, adverse childhood experiences, childhood IQ score, childhood self-control, and childhood health). For each characteristic, values were standardized to a T distribution (M=50, SD=10) with high scores reflecting increased risk (e.g. shorter-lived grandparents, lower childhood social class). Standardized values were summed to calculate the cumulative risk score. Thus, the expected cumulative risk level was 300. The graph shows that 2/3 of the slow-aging group had below this expected level of risk. In contrast, less than 1/3 of the fast-aging group did. **Panel B** graphs proportions of Study members with Biological Age 1 SD or more younger than the cohort mean, within 1 SD of the cohort mean, and 1 SD or more older than the cohort mean who were classified as high-risk on 0, 1, or 2-or-more of the 6 characteristics. High-risk classifications were for having short-lived grandparents (no grandparent survived past age 80 years), growing up in a low-social class family, exposure to 4 or more adverse childhood experiences, childhood IQ score ≤1 SD below the population mean (a score of 85 or below), childhood self-control score ≤1 SD below the population mean, and childhood health score ≤1 SD below the population mean. The graph shows that most Study members with younger Biological Ages had no high-risk classifications. In contrast, more than 40% of the Study members with older Biological Ages were classified as high-risk on multiple family and childhood characteristics.

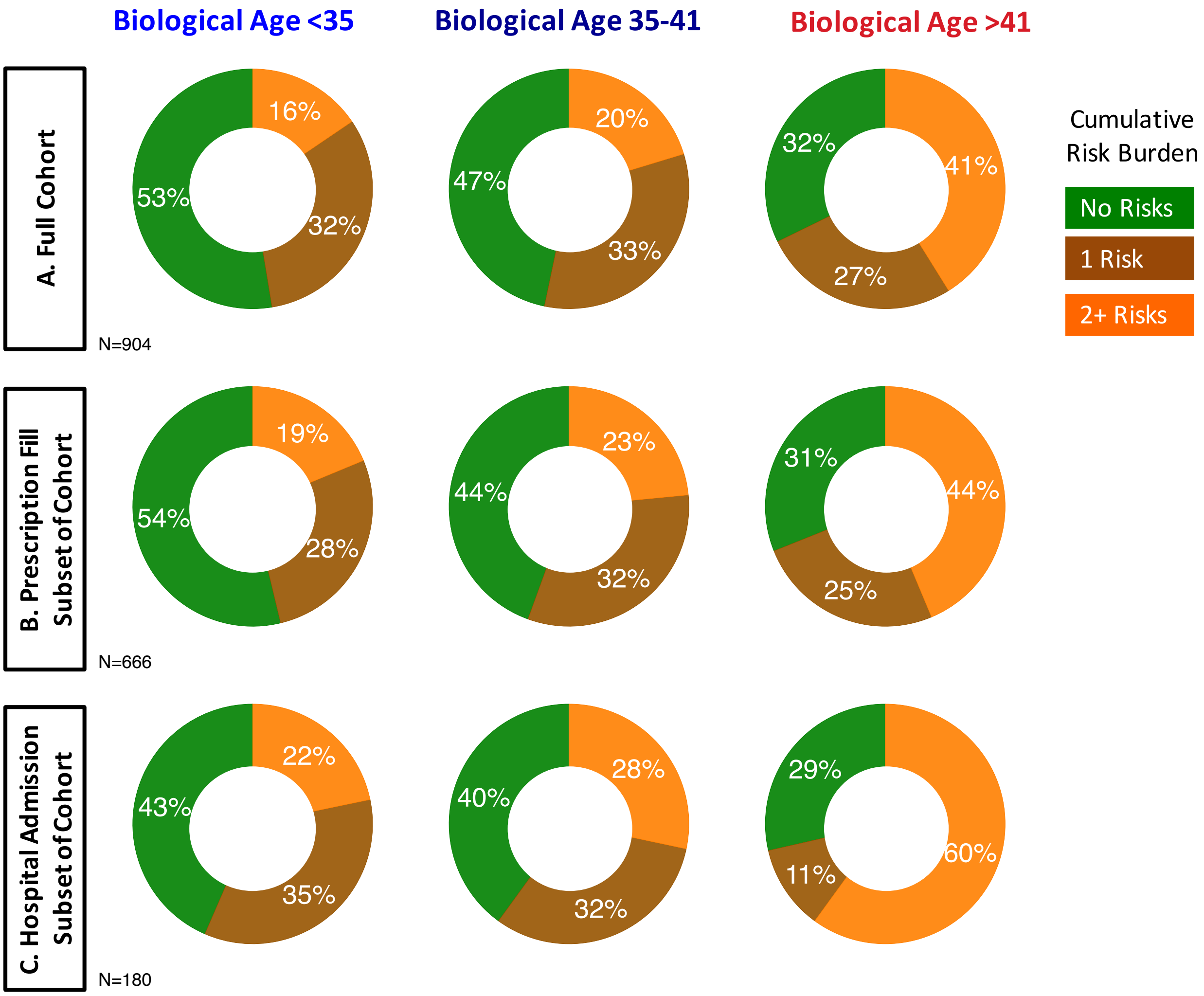

Supplemental Figure 4. Proportions of members with Biological Age <35, 35-41, and >41 classified as high-risk on 0, 1, or 2-or-more family and childhood characteristics based on contemporaneous assessments conducted in adulthood. Risk factors were having short-lived grandparents (no grandparent survived past age 80 years), retrospective report by the Study member that their parents held low-status occupations during the Study member's childhood, retrospective report of exposure to 4 or more adverse childhood experiences, not holding any educational credential, and being rated by an examining nurse as having low levels of the personality trait conscientiousness. **Panel A** graphs results for the full cohort. The pattern is the same as when risk was classified from assessments during childhood. Most slow-aging Study members were not classified as high-risk on any family or childhood characteristic. In contrast, more than 40% of the fast-aging Study members were classified as high-risk on multiple family and childhood characteristics. Panels B and C repeat the analysis for subsamples of cohort members with recent contacts with the health care system and who may reflect the population most accessible to recruitment into clinical trials. **Panel B** graphs results for Study members with a recent prescription fill. **Panel C** graphs results for Study members with a recent hospital admission (excluding for pregnancy-related services).

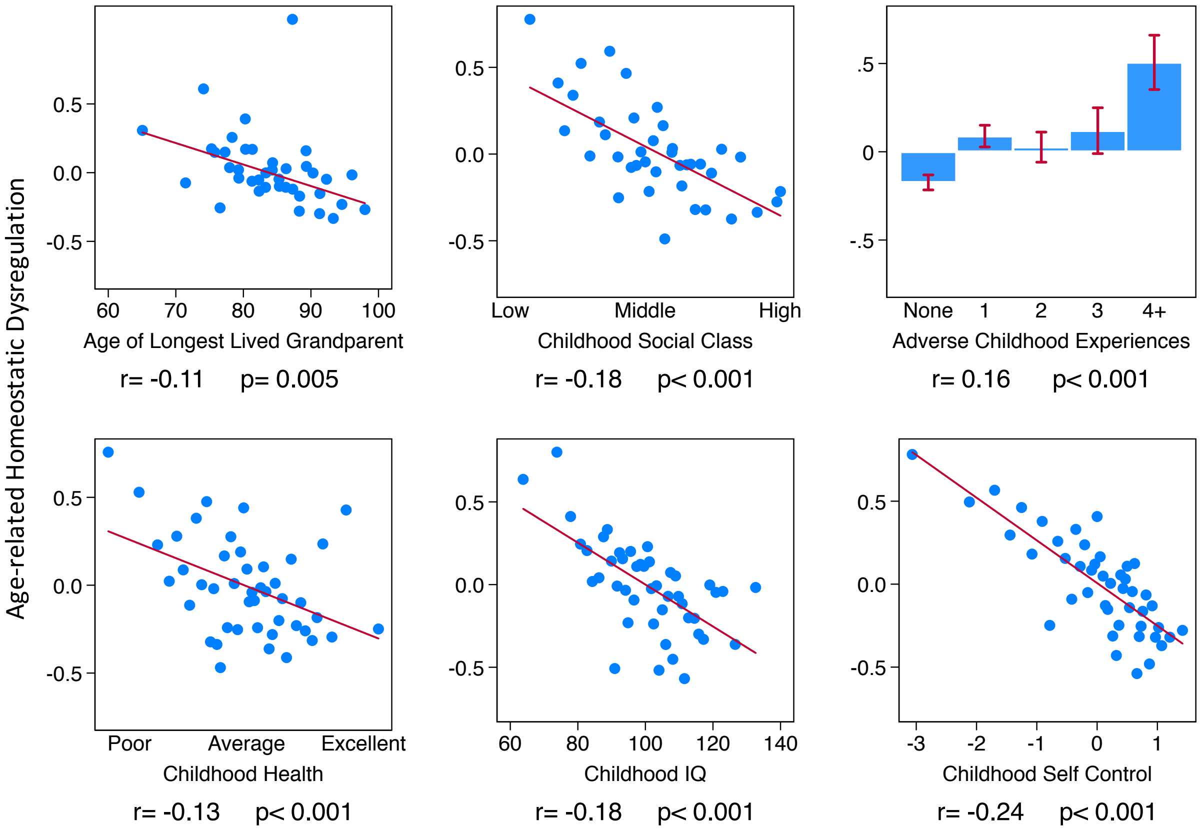

Supplemental Figure 5. Family and childhood characteristics are associated with Study members' age-related homeostatic dysregulation measured at chronological age 38 years. Figure cells graph associations between 6 family and childhood characteristics (x-axes) and Study members' Biological Age (y-axis). Age-related homeostatic dysregulation was measured using the methodology described by Cohen and colleagues *(Cohen et al. 2015; Li et al. 2015)* applied to the 18 Pace of Aging biomarkers. Age of longest-lived grandparent was measured from reports by Study members' parents. Childhood social class, exposure to adverse childhood experiences, childhood health, childhood IQ, and childhood self-control were assessed using previously established methodology applied to archival Dunedin Study records including exams and testing, reports by parents and teachers, clinician ratings, administrative records, and direct observations. Figures show "binned" scatterplots in which each plotted point reflects average x- and y-coordinates for "bins" of approximately 20 Study members. Regression lines and effect-size estimates were estimated from the original, un-binned data.

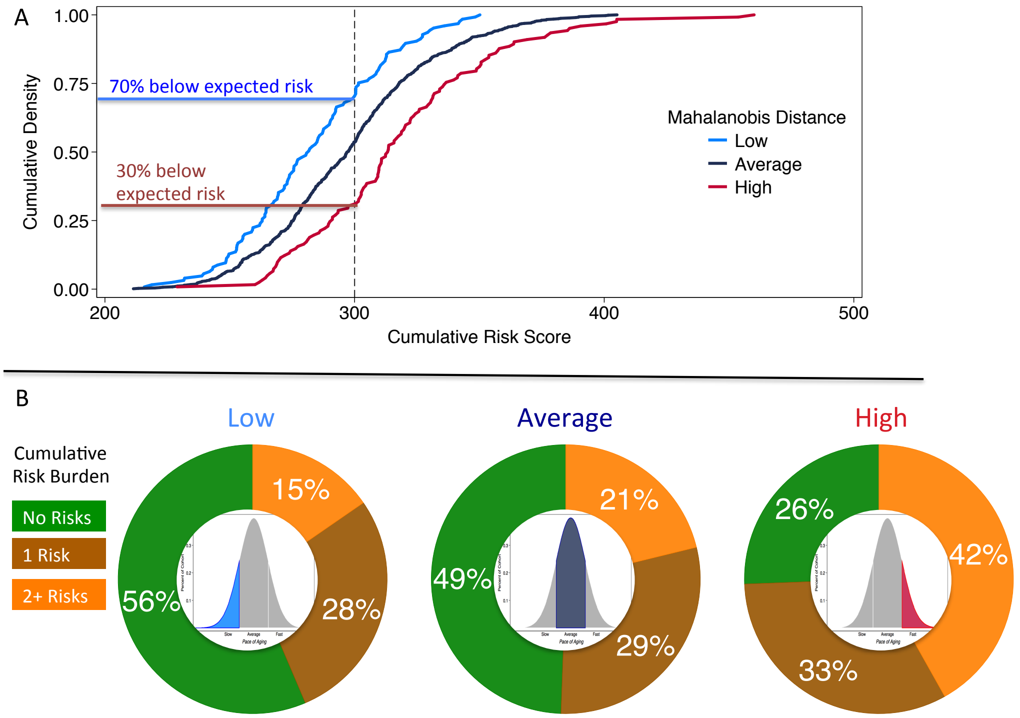

Supplemental Figure 6. Cumulative prospectively-assessed personal-history risks in Study members with low, average, and high levels of age-related homeostatic dysregulation. Panel A graphs density plots of cumulative risk scores for Study members with age-related homeostatic dysregulation scores 1 SD or more younger than the cohort mean (<35 years), within 1 SD of the cohort mean, and >1 SD or more older than the cohort mean. The cumulative risk score reflects total burden of risk across 6 personal history characteristics (grandparent longevity, family social class during childhood, adverse childhood experiences, childhood IQ score, childhood self-control, and childhood health). For each characteristic, values were standardized to a T distribution (M=50, SD=10) with high scores reflecting increased risk (e.g. shorter-lived grandparents, lower childhood social class). Standardized values were summed to calculate the cumulative risk score. Thus, the expected cumulative risk level was 300. The graph shows that 2/3 of the low-dysregulation group had below this expected level of risk. In contrast, less than 1/3 of the high-dysregulation group did. **Panel B** graphs proportions of Study members with age-related homeostatic dysregulation scores 1 SD or more younger than the cohort mean, within 1 SD of the cohort mean, and 1 SD or more older than the cohort mean who were classified as high-risk on 0, 1, or 2-or-more of the 6 characteristics. High-risk classifications were for having short-lived grandparents (no grandparent survived past age 80 years), growing up in a low-social class family, exposure to 4 or more adverse childhood experiences, childhood IQ score ≤1 SD below the population mean (a score of 85 or below), childhood self-control score ≤1 SD below the population mean, and childhood health score ≤1 SD below the population mean. The graph shows that most Study members with low age-related homeostatic dysregulation scores had no high-risk classifications. In contrast, more than 40% of the Study members with high age-related homeostatic dysregulation scores were classified as high-risk on multiple family and childhood characteristics.

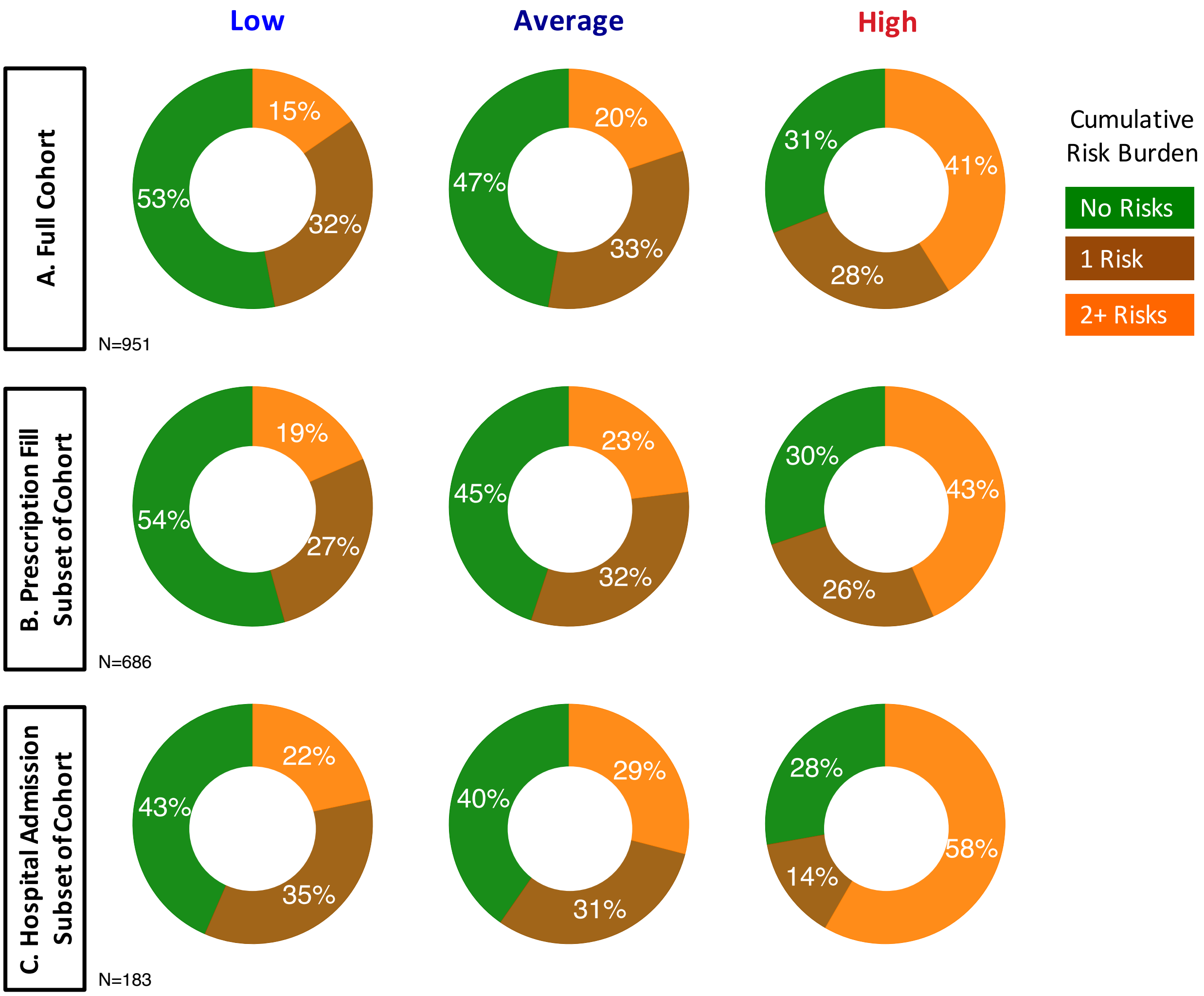

Supplemental Figure 7. Proportions of members with low, average, and high levels of age-related homeostatic dysregulation classified as high-risk on 0, 1, or 2-or-more family and childhood characteristics based on contemporaneous assessments conducted in adulthood. Risk factors were having short-lived grandparents (no grandparent survived past age 80 years), retrospective report by the Study member that their parents held low-status occupations during the Study member's childhood, retrospective report of exposure to 4 or more adverse childhood experiences, not holding any educational credential, and being rated by an examining nurse as having low levels of the personality trait conscientiousness. **Panel A** graphs results for the full cohort. The pattern is the same as when risk was classified from assessments during childhood. Most low-dysregulation Study members were not classified as high-risk on any family or childhood characteristic. In contrast, more than 40% of the high-dysregulation Study members were classified as high-risk on multiple family and childhood characteristics. Panels B and C repeat the analysis for subsamples of cohort members with recent contacts with the health care system and who may reflect the population most accessible to recruitment into clinical trials. **Panel B** graphs results for Study members with a recent prescription fill. **Panel C** graphs results for Study members with a recent hospital admission (excluding for pregnancy-related services).
